# A Clinical Review of the Connections Between Diabetes Mellitus, Periodontal Disease, and Cardiovascular Pathologies

**DOI:** 10.3390/biomedicines13092309

**Published:** 2025-09-20

**Authors:** Otilia Țica, Ioana Romanul, Gabriela Ciavoi, Vlad Alin Pantea, Ioana Scrobota, Lucian Șipoș, Cristian Marius Daina, Ovidiu Țica

**Affiliations:** 1 Cardiology Clinic, Emergency County Clinical Hospital of Bihor, 410165 Oradea, Romania; 2 Department of Dental Medicine, Faculty of Medicine and Pharmacy, University of Oradea, 410068 Oradea, Romania; gciavoi@uoradea.ro (G.C.); vlad_pantea@uoradea.ro (V.A.P.); ioana_scrobota@uoradea.ro (I.S.); lsipos@uoradea.ro (L.Ș.); 3 Department of Surgical Disciplines, Faculty of Medicine and Pharmacy, University of Oradea, 410073 Oradea, Romania; cdaina@uoradea.ro; 4 Department of Morphological Disciplines, Faculty of Medicine and Pharmacy, University of Oradea, 410073 Oradea, Romania; ovidiu.tica@gmail.com; 5 Department of Pathology, Emergency County Clinical Hospital of Bihor, 410165 Oradea, Romania

**Keywords:** diabetes mellitus, cardiovascular disease, endothelial dysfunction, insulin resistance, atherosclerosis, periodontal disease

## Abstract

**Background**: Diabetes mellitus (DM), periodontal disease (PD), and cardiovascular disease (CVD) are highly prevalent global health conditions with overlapping pathophysiological mechanisms. Emerging evidence suggests a bidirectional and synergistic relationship among them, driven by chronic inflammation, immune dysregulation, oxidative stress, and microbial dysbiosis. **Objective**: This review synthesizes current literature on the interconnectedness of DM, PD, and CVD, emphasizing shared molecular pathways, clinical implications, and opportunities for integrated management. **Methods**: A systematic review and narrative synthesis of recent clinical trials, observational studies, and multi-omics investigations was conducted to explore the mechanisms linking these three conditions. A structured literature search was performed across PubMed, Scopus, and Web of Science from database inception until 30 June 2025. Key findings were contextualized within systems biology, precision medicine, and real-world clinical strategies. **Results**: DM exacerbates periodontal inflammation and accelerates tissue destruction via hyperglycemia-induced inflammatory mediators, while periodontitis worsens glycemic control and insulin resistance. Both conditions independently elevate cardiovascular risk, and their co-occurrence significantly amplifies the incidence of adverse cardiovascular events. Shared biomarkers such as Interleukin (IL)-6, Tumor Necrosis Factor (TNF)-α, and CRP, as well as overlapping genetic and epigenetic signatures, underscore a common inflammatory axis. Periodontal therapy has demonstrated modest but meaningful benefits on glycemic control and endothelial function, while cardiometabolic therapies (e.g., statins, Glucagon-Like Peptide (GLP-1) receptor agonists, SGLT2 inhibitors) show potential to improve periodontal outcomes. Probiotics, microbiome-targeted therapies, and AI-based risk models are emerging as future tools. **Conclusions**: DM, PD, and CVD form a mutually reinforcing triad mediated by systemic inflammation and metabolic dysregulation. Integrated, multidisciplinary care models and precision health strategies are essential to address this inflammatory burden and improve long-term outcomes. Further large-scale interventional trials and mechanistic human studies are needed to establish causal links and optimize combined therapeutic approaches.

## 1. Introduction

Diabetes mellitus (DM), periodontal disease (PD), and cardiovascular disease (CVD) represent three major global health concerns that not only share common risk factors but also appear to be pathophysiologically interconnected. DM currently affects over 500 million individuals globally and is expected to rise steadily due to aging populations and lifestyle changes [1]. Periodontal disease, a chronic inflammatory condition of the gums and supporting tissues, affects approximately 50% of adults worldwide, with severe forms impacting up to 10% [2]. CVD remains the leading cause of morbidity and mortality worldwide, responsible for an estimated 18 million deaths annually [3,4].

Emerging evidence underscores a bidirectional relationship between DM and PD, wherein each condition exacerbates the other. Simultaneously, both DM and PD have been independently associated with increased cardiovascular risk, hinting at shared systemic inflammatory pathways. Chronic inflammation, immune dysregulation, oxidative stress, and endothelial dysfunction are recurrent themes across these conditions [5]. In this comprehensive systematic review, we aim to synthesize current clinical and mechanistic evidence linking diabetes mellitus, periodontal disease, and cardiovascular disease, with particular emphasis on their overlapping mechanisms of inflammation, metabolic dysregulation, and microbial dysbiosis. The main objectives are: (i) to synthesize current clinical and mechanistic evidence connecting these three conditions, (ii) to highlight the shared inflammatory, metabolic, and microbial pathways that underpin their interactions, and (iii) to explore translational, therapeutic, and multidisciplinary strategies for integrated management. Unlike previous reviews that have examined these associations in isolation, our work integrates data across the three axes to highlight their triangular interplay. By systematically presenting clinical, mechanistic, and therapeutic perspectives, this review contributes to the field by framing periodontal disease as a cardiometabolic risk enhancer and by outlining future translational opportunities, including multi-omics, AI-based tools, and integrated care models.

## 2. Materials and Methods

We conducted this review in accordance with the Preferred Reporting Items for Systematic Reviews and Meta-Analyses (PRISMA) 2020 statement [6]. The protocol was not registered in PROSPERO because this was designed as a narrative clinical review; however, the systematic framework was applied to ensure transparency and reproducibility.

### 2.1. Information Sources and Search Strategy

The literature search was conducted with the explicit aim of identifying studies that addressed at least one of the following: mechanistic links between DM, PD, and CVD; shared biomarkers or inflammatory pathways; and clinical or therapeutic strategies relevant to their integrated management.”

A comprehensive literature search was carried out across PubMed/MEDLINE, Scopus, and Web of Science databases, covering the period from database inception until 30 June 2025. The search combined the following keywords and Medical Subject Headings (MeSH): “periodontal disease”, “diabetes mellitus”, “cardiovascular disease”, “atherosclerosis”, “inflammation”, “oral-systemic connection”, and “endothelial dysfunction.” Boolean operators (AND/OR) were used to refine the strategy.

In addition, the reference lists of relevant articles and recent reviews were screened manually to identify further eligible studies.

### 2.2. Eligibility Criteria

Studies were included if they met the following criteria: (i) peer-reviewed articles published in English; (ii) human clinical studies (observational studies, randomized controlled trials, or meta-analyses); and (iii) mechanistic or translational studies directly linking DM, PD, and/or CVD through inflammatory, microbial, or metabolic pathways.

We excluded (i) non-peer-reviewed publications, case reports, letters, or conference abstracts without full data; (ii) purely animal or in vitro studies unless they offered direct mechanistic insight relevant to humans; and (iii) articles without accessible full texts.

### 2.3. Study Selection and Data Extraction

After duplicate removal, two reviewers (O.T. and I.R.) independently screened titles and abstracts, followed by full-text evaluation for potentially eligible records. Data were extracted using a predefined form, collecting information on study design, sample characteristics, outcomes, biomarkers, and therapeutic implications. Any disagreements were resolved through discussion with a third author (O.A.T).

The database searches identified a total of 2145 records. After removal of 345 duplicates, 1800 records were screened by title and abstract. Of these, 1420 were excluded as not relevant to the scope of this review. The remaining 380 full-text articles were retrieved and assessed for eligibility. Following full-text review, 270 studies were excluded for the following reasons: insufficient mechanistic or clinical relevance (n = 145), case reports or letters only (n = 68), non-English language (n = 32), or inaccessible full text (n = 25). Ultimately, 110 studies met all inclusion criteria and were incorporated into the qualitative synthesis.

### 2.4. Methodological Note

Given the narrative clinical review design, no new clinical or laboratory data were collected. All searches, screening, and data extraction were performed manually by two independent reviewers (O.T. and I.R.), with disagreements resolved by a third author (O.A.T.). No automated text-mining, keyword scraping, or AI-based tools were used in this review. The synthesis represents a qualitative integration of evidence. No statistical pooling (meta-analysis) was conducted; findings were integrated through narrative synthesis.

## 3. Diabetes Mellitus and Periodontal Disease: Bidirectional Interactions

This review synthesized evidence from 110 eligible studies that investigated the relationships between diabetes mellitus (DM), periodontal disease (PD), and cardiovascular disease (CVD). The findings are presented in four domains corresponding to the three established axes and their triangular interaction.

The relationship between diabetes mellitus and periodontal disease has been extensively studied and is widely regarded as bidirectional. Patients with poorly controlled diabetes exhibit a higher prevalence and severity of periodontal disease, while those with periodontitis often present with poorer glycaemic control [7].

This review synthesized evidence from 110 eligible studies identified through our structured literature search across PubMed, Scopus, and Web of Science. The included works comprised observational studies, randomized controlled trials, mechanistic analyses, and meta-analyses, all of which addressed at least one of the predefined objectives of this review. For clarity, the findings are presented systematically according to the three established disease axes and their triangular interaction: (i) the relationship between diabetes mellitus and periodontal disease (DM–PD), (ii) the association between periodontal disease and cardiovascular disease (PD–CVD), (iii) the well-established axis of diabetes mellitus and cardiovascular disease (DM–CVD), and (iv) the triangular relationship that integrates all three conditions.

### 3.1. Pathways and Clinical Outcomes of the Diabetes–Periodontitis Interaction

The literature consistently supports a bidirectional association between DM and PD, wherein hyperglycemia-driven inflammation worsens periodontal outcomes, while periodontal disease contributes to poor glycemic control.

#### 3.1.1. Inflammatory Pathways

One of the primary mechanisms underpinning this association involves systemic inflammation. Hyperglycaemia leads to increased production of advanced glycation end-products (AGEs), which bind to RAGE (receptors for AGEs) on immune cells, enhancing the secretion of pro-inflammatory cytokines such as TNF-α, IL-1β, and IL-6 [8]. These cytokines exacerbate periodontal tissue destruction and impair wound healing in gingival tissues.

These inflammation-related mechanisms are supported by clinical studies showing significant reductions in HbA1c and CRP following periodontal therapy in T2DM patients [9], and in cross-sectional comparisons demonstrating higher HbA1c in diabetic individuals with periodontitis compared to those without.

**Figure 1:**
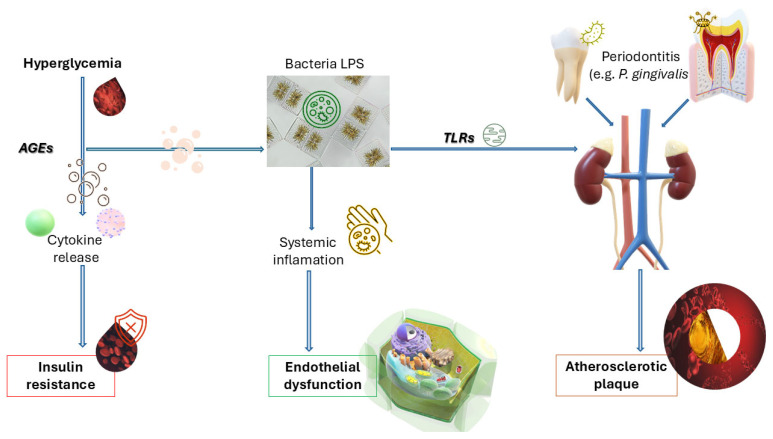
Biological cascade from hyperglycemia to atherosclerosis via periodontitis. Biological cascade from hyperglycemia to atherosclerosis via periodontitis. The flowchart illustrates the connections between hyperglycemia, bacterial lipopolysaccharide (LPS) from *Porphyromonas gingivalis*, advanced glycation end-products (AGEs), and toll-like receptor (TLR) activation, and their downstream effects on insulin resistance, systemic inflammation, and atherosclerotic plaque development. Evidence supporting these pathways has been reported in mechanistic and clinical studies linking hyperglycaemia, periodontal inflammation, and vascular dysfunction [4,5,7]. Abbreviation: AGEs—Advanced Glycation End-products: harmful compounds formed from sugar–protein reactions; LPS—Lipopolysaccharide: a bacterial endotoxin that triggers immune responses; *P. gingivalis*—*Porphyromonas gingivalis*: a key periodontal pathogen; TLRs—Toll-like Receptors: receptors involved in pathogen recognition and immune activation.

The interplay between metabolic dysregulation and microbial inflammation is depicted in Figure 1, which outlines how hyperglycaemia-induced AGEs, bacterial LPS (notably from *P. gingivalis*), and TLR activation synergistically contribute to insulin resistance, systemic cytokine release, and ultimately, the progression of vascular inflammation and atherogenesis.

The figures included in this review are evidence-based conceptual models designed to synthesize published findings into accessible visual frameworks. Each pathway or mediator depicted is directly supported by peer-reviewed studies, systematic reviews, or consensus reports, as detailed in the revised figure legends. Thus, the figures are not speculative but rather serve as integrative summaries of current mechanistic and clinical evidence.

In periodontal disease, inflammation is primarily localized to the gingival and periodontal tissues, leading to connective tissue breakdown and alveolar bone loss. In diabetes mellitus, chronic low-grade inflammation affects pancreatic islets, liver, and adipose tissue, contributing to impaired insulin secretion and systemic insulin resistance. Cardiovascular disease is characterized by inflammation within the vascular endothelium and atherosclerotic plaques, where activated immune cells and cytokines promote plaque instability. Adipose tissue is increasingly recognized as a key metabolic and inflammatory organ in DM. Recent studies [10,11] show that adipose tissue in T2D exhibits inflammatory cell infiltration, altered adipokine profiles, and contributes to systemic inflammation, which links PD and CVD.

Periodontal pathogens like *Porphyromonas gingivalis* also contribute to systemic inflammation by stimulating toll-like receptors (TLRs) and promoting the release of systemic inflammatory mediators, which worsen insulin resistance [12]. This vicious cycle amplifies both periodontal and diabetic pathologies. These inflammatory processes provide a mechanistic rationale for the clinical outcomes described in Section 3.1.2 and Section 3.1.3, where improved glycemic control (reflected by HbA1c reduction) following periodontal therapy can be partly explained by reductions in systemic cytokines such as IL-6 and TNF-α, and by improvements in endothelial function.

#### 3.1.2. Clinical Evidence and Outcomes

Meta-analyses have shown that individuals with diabetes are 2 to 3 times more likely to develop periodontitis than those without diabetes [13]. Conversely, interventional trials demonstrate that nonsurgical periodontal therapy (NSPT) improves glycemic control, with reported reductions in HbA1c ranging from 0.3% to 0.6% over a 3- to 6-month period [4].

A recent randomized controlled trial involving over 1000 patients with type 2 diabetes reported that periodontal therapy was associated with significant improvements in HbA1c, independent of medication changes, highlighting the systemic benefits of oral healthcare [14].

For instance, Hayashi et al. [15]. observed that non-surgical periodontal treatment not only improved periodontal health but also reduced HbA1c levels and systemic markers of inflammation including TNF-α, linking improved glycemic control to reduced inflammatory burden.

#### 3.1.3. Glycemic Control as a Modifying Factor

Glycemic control directly influences periodontal outcomes. Patients with poorly controlled diabetes (HbA1c > 8%) exhibit more rapid periodontal destruction, including alveolar bone loss, compared to those with better metabolic control [16]. This suggests that strict glycemic regulation should be a cornerstone in periodontal management for diabetic individuals.

### 3.2. Periodontal Disease and Cardiovascular Disease: Inflammation as a Common Denominator

Multiple studies demonstrate that periodontal disease is linked with increased cardiovascular risk via bacteremia, systemic inflammation, and endothelial dysfunction.

The connection between periodontal disease (PD) and cardiovascular disease (CVD) has garnered significant attention in recent years, with increasing evidence supporting a causal or at least contributory relationship. The hypothesis centers on shared mechanisms of chronic inflammation, bacteremia, and immune dysregulation, which link oral infections to systemic vascular pathologies [17].

#### 3.2.1. Epidemiological Associations

Multiple observational studies and meta-analyses have demonstrated a consistent association between periodontitis and increased cardiovascular risk, including coronary artery disease (CAD), myocardial infarction (MI), and stroke [18,19]. A large-scale cohort study found that individuals with severe periodontitis had a 1.6-fold higher risk of major adverse cardiovascular events (MACE), even after adjusting for traditional risk factors such as smoking, diabetes, and hypertension [20].

Furthermore, a meta-analysis concluded that periodontitis increases the risk of future cardiovascular events by 20% to 25%, particularly in individuals with comorbid conditions [21].

#### 3.2.2. Mechanistic Insights: From Mouth to Heart

The underlying pathophysiological mechanisms that connect PD and CVD involve both direct and indirect pathways:

##### Bacteremia and Endothelial Dysfunction

Periodontal pathogens such as *Porphyromonas gingivalis*, *Aggregatibacter actinomycetemcomitans*, and *Tannerella forsythia* can enter the systemic circulation during daily oral activities or dental procedures. These microbes have been detected in atherosclerotic pla ques, suggesting a direct role in vascular pathology [22]. While acute bacteremia may rarely precipitate sepsis, the more clinically relevant mechanism in the DM–PD–CVD axis is chronic low-grade bacteremia, which sustains systemic inflammation and contributes to progressive atherosclerosis and plaque instability [9,15,23,24]. Observations of periodontal pathogens in atheromatous plaques and observations of monocyte adhesion, endothelial disruption, and immune activation [24] further support the hypothesis that chronic low-grade bacteremia contributes to atherosclerotic progression rather than merely acute sepsis. A recent study confirmed the presence of DNA from *P. gingivalis* and its lipopolysaccharide (LPS) in coronary artery samples obtained from patients undergoing coronary artery bypass grafting [25].

This translocation triggers an inflammatory cascade, leading to endothelial activation, increased expression of adhesion molecules (e.g., Vascular Cell Adhesion Molecule (VCAM)-1, Intercellular Adhesion Molecule (ICAM)-1), and subsequent leukocyte infiltration—hallmarks of early atherogenesis.

**Figure 2:**
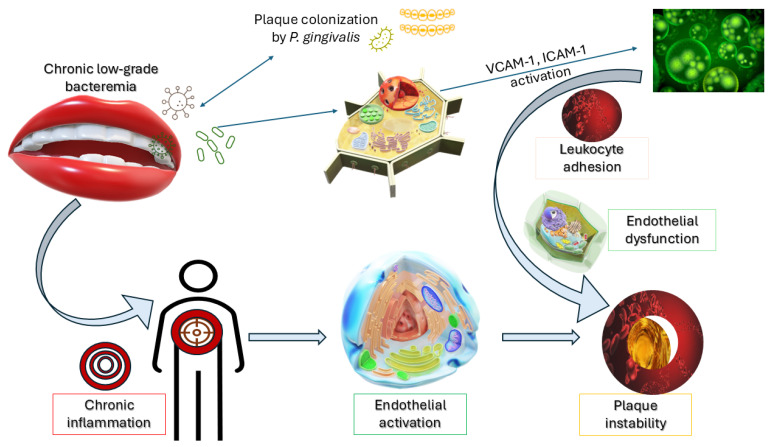
Pathway linking bacteremia and atherosclerotic plaque instability. The diagram illustrates how periodontal pathogens such as *Porphyromonas gingivalis* enter the circulation, activate endothelial adhesion molecules (VCAM-1, ICAM-1), and promote leukocyte recruitment, systemic inflammation, and plaque vulnerability. While acute bacteremia can rarely result in sepsis, the more clinically relevant pathway is chronic low-grade bacteremia, which sustains vascular inflammation and contributes to atherosclerosis. This figure is based on evidence showing the detection of periodontal bacteria in coronary plaques and their role in endothelial dysfunction and plaque vulnerability [12,26]. Abbreviation: ICAM-1—Intercellular Adhesion Molecule-1: another key adhesion molecule in vascular inflammation; *P. gingivalis*—*Porphyromonas gingivalis*: periodontal bacterium implicated in systemic inflammation; VCAM-1—Vascular Cell Adhesion Molecule-1: facilitates leukocyte adhesion to endothelial cells.

As illustrated in Figure 2, the translocation of *P. gingivalis* into systemic circulation leads to endothelial activation and the upregulation of adhesion molecules such as VCAM-1 and ICAM-1. This cascade fosters leukocyte recruitment, chronic inflammation, and increased vulnerability of atherosclerotic plaques—offering a visual summary of the oral-systemic interface in vascular pathology.

##### Systemic Inflammation and Cytokine Release

Chronic periodontal infection results in the sustained release of inflammatory mediators such as interleukin-6 (IL-6), tumor necrosis factor-alpha (TNF-α), and C-reactive protein (CRP), which circulate systemically and contribute to vascular inflammation and plaque instability [27]. Elevated CRP levels have been independently associated with both periodontitis and cardiovascular events, reinforcing the inflammation-driven model of CVD [28].

In a recent prospective cohort study of patients with type 2 diabetes, individuals with high periodontal inflammatory burden demonstrated significantly elevated serum IL-6 and CRP levels, which correlated with increased coronary artery calcification scores [25]. This primary patient-based study provides clinical evidence linking systemic inflammatory mediators from PD with subclinical atherosclerosis [29].

#### 3.2.3. Clinical Impact of Periodontal Treatment on Cardiovascular Outcomes

Several interventional studies have examined whether treating periodontitis may reduce systemic inflammation and improve cardiovascular health. In a randomized controlled trial including patients with moderate-to-severe periodontitis and established cardiovascular disease, intensive periodontal therapy significantly improved brachial artery flow-mediated dilation (FMD) at four weeks and was associated with reductions in circulating CRP and IL-6 levels [17,30,31]. These biomarker changes indicate a short-term anti-inflammatory effect, but it is important to clarify that lowering inflammatory markers does not necessarily equate to a complete resolution of systemic inflammation.

Although long-term cardiovascular endpoints remain to be conclusively proven, these findings suggest that periodontal treatment may serve as a complementary strategy for managing systemic inflammation in high-risk cardiovascular patients.

#### 3.2.4. The Oral-Systemic Inflammatory Axis

Emerging research underscores the concept of an “oral-systemic inflammatory axis,” where chronic oral inflammation acts as a reservoir for systemic immune activation. A consensus report published in the *European Heart Journal* emphasized the importance of the oral–systemic inflammatory axis in a systems biology framework, proposing that PD may serve as both a marker and a mediator of cardiovascular risk, particularly in individuals with preexisting metabolic disturbances [26].

Additionally, oral dysbiosis—disruption of the normal oral microbiota—has been implicated in endothelial dysfunction and systemic inflammation. Studies using shotgun metagenomic sequencing have found increased abundance of pro-atherogenic bacteria in the oral cavities of patients with coronary artery disease [32].

### 3.3. Diabetes and Cardiovascular Disease: A Well-Established Axis

The relationship between DM and CVD is robust and extensively documented, with hyperglycemia, insulin resistance, endothelial dysfunction, and dyslipidemia as central mediators. Cardiovascular complications are the leading cause of morbidity and mortality in individuals with diabetes, accounting for more than 65% of deaths in this population [33]. The connection is multifactorial, involving hyperglycemia, insulin resistance, endothelial dysfunction, dyslipidemia, and systemic inflammation.

#### 3.3.1. Hyperglycemia and Endothelial Dysfunction

Chronic hyperglycemia plays a pivotal role in the development of cardiovascular pathology by impairing endothelial function. Elevated glucose levels promote the generation of reactive oxygen species (ROS), reduce nitric oxide bioavailability, and increase the formation of advanced glycation end-products (AGEs) [34]. These biochemical changes lead to increased vascular permeability, leukocyte adhesion, and smooth muscle proliferation—hallmarks of atherosclerosis.

In a recent study using transcriptomic profiling of human coronary artery endothelial cells (HCAECs) obtained from patients with poorly controlled diabetes, investigators reported significant upregulation of both pro-inflammatory and pro-thrombotic gene networks [35]. Specifically, increased expression of IL-6, TNF-α, and NF-κB–related signaling pathways was observed, alongside enhanced transcription of pro-thrombotic mediators such as tissue factor (F3), plasminogen activator inhibitor-1 (PAI-1/SERPINE1), and von Willebrand factor (vWF). These findings provide direct molecular evidence that hyperglycemia induces endothelial dysfunction by simultaneously driving inflammation and thrombosis in the vascular wall.

#### 3.3.2. Insulin Resistance, Dyslipidemia, and Atherosclerosis

Insulin resistance, central to the development of type 2 diabetes, also contributes to atherogenesis through multiple mechanisms. It leads to increased free fatty acid release, hepatic overproduction of very-low-density lipoprotein (VLDL), and reduced high-density lipoprotein (HDL), promoting an atherogenic lipid profile [36]. At the molecular level, these changes are associated with activation of NF-κB signaling and upregulation of adhesion molecules such as ICAM-1 and VCAM-1, which facilitate leukocyte recruitment to the endothelium. Pro-inflammatory cytokines including IL-6 and TNF-α further amplify vascular inflammation, while impaired endothelial nitric oxide synthase (eNOS) activity reduces nitric oxide (NO) bioavailability. In parallel, pro-thrombotic mediators such as plasminogen activator inhibitor-1 (PAI-1) and tissue factor are upregulated, enhancing thrombosis risk. Together, these molecular alterations explain how insulin resistance promotes endothelial dysfunction, inflammation, and thrombotic complications in patients with diabetes and cardiovascular disease. A review noted that insulin resistance is associated with increased carotid intima-media thickness and coronary artery calcification, both strong predictors of future cardiovascular events [37].

#### 3.3.3. Role of HbA1c and Glycemic Variability

Hemoglobin A1c (HbA1c) is a well-established marker for long-term glycemic control and cardiovascular risk. Elevated HbA1c is directly correlated with an increased incidence of myocardial infarction, stroke, and heart failure. A meta-analysis found that each 1% increase in HbA1c was associated with an 18% increase in cardiovascular events [38].

However, emerging data suggest that glycemic variability—fluctuations in blood glucose levels—is an independent risk factor for cardiovascular outcomes, possibly due to oxidative stress and vascular inflammation. This was demonstrated in a review examining continuous glucose monitoring data in high-risk diabetic populations [39].

#### 3.3.4. Evidence from Cardiovascular Outcome Trials (CVOTs)

Several large-scale cardiovascular outcome trials (CVOTs) have transformed our understanding of the interplay between diabetes treatment and cardiovascular risk:

SGLT2 inhibitors: Trials such as EMPA-REG OUTCOME (empagliflozin) [40] and DAPA-HF (dapagliflozin) [41] have shown significant reductions in hospitalization for heart failure and cardiovascular mortality [42,43].

GLP-1 receptor agonists: Liraglutide (LEADER trial) [44] and semaglutide (SUSTAIN-6) [45] reduced the incidence of major adverse cardiovascular events (MACE) in type 2 diabetic patients [46].

A comprehensive meta-analysis covering 14 CVOTs concluded that both SGLT2 inhibitors and GLP-1 receptor agonists offer consistent cardiovascular protection in diabetic populations, particularly against heart failure [47] and atherosclerotic events [48,49].

#### 3.3.5. Genetic and Epigenetic Factors

Recent studies have uncovered shared genetic and epigenetic pathways between DM and CVD. A multi-omics integrative analysis identified common polymorphisms in inflammatory genes such as *IL6*, *TNF*, and *NFKB1*, which modulate both glycemic control and cardiovascular risk [50].

Moreover, epigenetic modifications like DNA methylation and histone acetylation have been implicated in the regulation of pro-inflammatory and metabolic pathways. These findings open new avenues for precision medicine targeting the shared biology of diabetes and cardiovascular disease.

### 3.4. Triangular Relationship: DM, PD, and CVD—A Shared Inflammatory Burden

Emerging evidence indicates that DM, PD, and CVD form a mutually reinforcing triad, underpinned by systemic inflammation, oxidative stress, microbial dysbiosis, and shared genetic susceptibilities.

While the individual links between diabetes mellitus (DM), periodontal disease (PD), and cardiovascular disease (CVD) are well documented, growing evidence suggests these three conditions form a mutually reinforcing triad mediated by chronic low-grade systemic inflammation, oxidative stress, immune dysregulation, and microbial dysbiosis. Understanding this triangular interplay is essential for advancing both pathophysiological insights and integrated clinical management strategies.

#### 3.4.1. Systems Biology Perspective

Systems biology approaches have been instrumental in delineating the interconnectedness of DM, PD, and CVD. A 2022 integrative omics study analyzed blood and tissue transcriptomes from patients with co-existing diabetes and periodontitis and found overlapping gene expression signatures related to inflammation, innate immunity, and endothelial activation [51]. Pathways involving nuclear factor (NF)-κB signaling, Toll-like receptor pathways, and oxidative phosphorylation were especially prominent in patients who later developed cardiovascular events.

Similarly, multi-omics network modeling revealed common nodes—such as IL-6, TNF-α, and ICAM-1—across the three disease states, underscoring the pivotal role of inflammation in driving systemic disease progression [52].

#### 3.4.2. Shared Biomarkers: Inflammatory and Immune Mediators

The inflammatory burden in DM, PD, and CVD arises from distinct yet overlapping tissue sites. In periodontal disease, gingival and periodontal connective tissues are the primary sites of chronic inflammation. In diabetes mellitus, inflammation occurs not only in pancreatic islets but also in adipose tissue and the liver, where it drives systemic insulin resistance. In cardiovascular disease, the vascular endothelium and atherosclerotic plaques serve as key inflammatory niches, where leukocyte infiltration and cytokine release accelerate plaque instability.

Several biomarkers consistently emerge across studies investigating DM, PD, and CVD [53]. Notably:

- C-reactive protein (CRP): Elevated in all three conditions; serves as a marker for systemic inflammation and cardiovascular risk [54].
- Interleukin-6 (IL-6): Linked to insulin resistance, periodontal inflammation, and endothelial dysfunction [55].
- Tumor necrosis factor-alpha (TNF-α): Involved in pancreatic β-cell dysfunction, periodontal tissue degradation, and atherosclerosis [56].
- Fibrinogen: A coagulation factor elevated in chronic inflammation and predictive of cardiovascular events [21].

Recent studies [57,58] confirm that in type 2 diabetes pancreatic islets undergo low-grade but persistent inflammation, with macrophage infiltration and activation of NF-κB and IL-1β signaling contributing to β-cell dysfunction. The single-cell transcriptomics work [10] further clarifies that pancreatic duct/duct-acinar cells express pro-inflammatory chemokines (e.g., CXCL9, CXCL10, etc.), suggesting that non-β endocrine cells also contribute to islet inflammation.

L-6 and TNF-α act as upstream drivers of insulin resistance and endothelial activation, amplifying vascular inflammation. NF-κB serves as a central transcriptional hub integrating microbial and metabolic signals [59]. Within the DM–PD–CVD triad, NF-κB activation drives pro-inflammatory cytokine release (IL-6, TNF-α), endothelial adhesion molecule expression (ICAM-1, VCAM-1), and amplifies systemic low-grade inflammation. While NF-κB is indeed implicated in numerous other chronic diseases, in this context it functions as a pivotal convergence point linking periodontal dysbiosis, hyperglycemia, and vascular dysfunction. CRP, produced in the liver in response to IL-6, is not only a marker but also an active participant in endothelial dysfunction, promoting monocyte adhesion and plaque progression. Together, these pathways highlight endothelial dysfunction and systemic cytokine signaling as pivotal mechanisms that unify local periodontal inflammation with metabolic and cardiovascular pathology.

Key molecular pathways operate across all three disease states. IL-6 and TNF-α promote insulin resistance in adipose tissue, impair periodontal tissue repair, and drive vascular inflammation. NF-κB functions as a transcriptional regulator that integrates microbial LPS from periodontal pathogens with metabolic stress signals, leading to sustained cytokine release in gingival, pancreatic, and vascular cells [60]. CRP, produced in the liver under IL-6 stimulation, actively contributes to endothelial dysfunction by enhancing leukocyte adhesion. NLRP3 inflammasome activation [61] and IL-1β release [62] amplify tissue destruction in periodontal pockets, β-cell apoptosis in pancreatic islets, and plaque instability in atherosclerosis [63]. Collectively, these circuits converge on endothelial dysfunction, which emerges as the key link between local periodontal inflammation, systemic metabolic dysregulation, and cardiovascular complications.

These mechanistic interactions have clear clinical implications. A prospective cohort study demonstrated that individuals with high levels of both IL-6 and CRP, along with moderate-to-severe periodontitis and type 2 diabetes, had a 2.3-fold increased risk of major adverse cardiovascular events compared to individuals with none of these risk factors [54].

**Figure 3:**
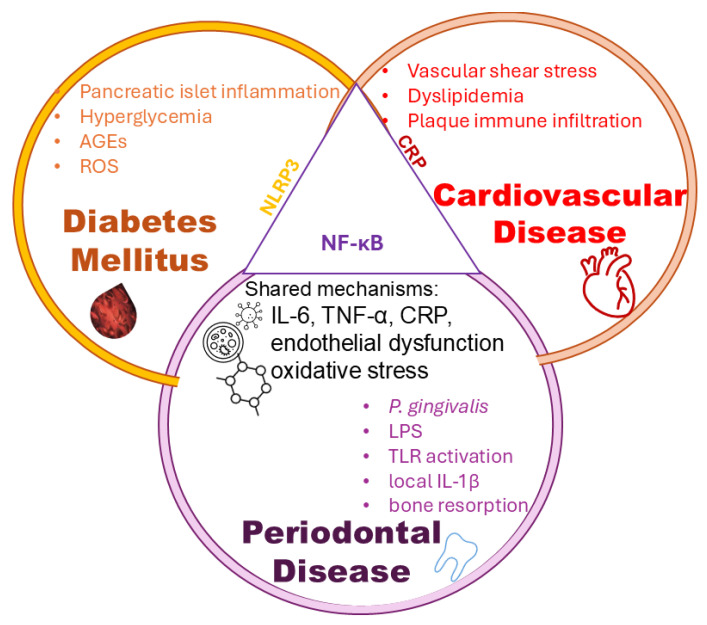
Shared inflammatory mechanisms linking diabetes, cardiovascular disease, and periodontal disease. A Venn diagram illustrates overlapping molecular pathways among DM, PD, and CVD. The shared circuits include IL-6, TNF-α, CRP, NF-κB, NLRP3 inflammasome activation, IL-1β signaling, oxidative stress, and endothelial dysfunction, which together drive systemic inflammation and cardiovascular risk. Evidence supporting these pathways is drawn from recent mechanistic and clinical studies. These mediators have been consistently reported as common drivers of systemic inflammation across the three conditions [54,55,64]. Abbreviations: CRP—C-reactive Protein; IL—Interleukin; NF-κB—Nuclear Factor kappa-light-chain-enhancer of activated B cells; NLRP3—NOD-like Receptor Family Pyrin domain-containing 3 (inflammasome); ROS—Reactive Oxygen Species; TNF-α—Tumor Necrosis Factor-alpha TLR—Toll-Like Receptor.

These overlapping pathophysiological mechanisms can be visually represented through a systems-level view of shared inflammatory mediators among diabetes mellitus, periodontal disease, and cardiovascular disease. Figure 3 illustrates the intersection of these three conditions, highlighting key molecules such as IL-6, TNF-α, CRP, and the roles of oxidative stress and endothelial dysfunction as central drivers of disease synergy.

Although inflammatory mediators such as IL-6, TNF-α, and CRP are elevated across all three conditions, endothelial dysfunction and systemic cytokine signaling represent central converging mechanisms, linking local periodontal inflammation and metabolic dysregulation to cardiovascular complications. The vascular endothelium itself is a primary site of inflammation in both CVD and the DM-PD axis. A recent study [65] reviewed how periodontal disease correlates with endothelial dysfunction (measured by markers such as flow-mediated dilation, VCAM/ICAM), and how periodontal therapy reduces endothelial inflammatory biomarkers. Such evidence strengthens the argument that endothelial tissue is a common denominator across all three diseases.

Key molecular pathways such as NF-κB, NLRP3 inflammasome activation, and IL-1β signaling are consistently implicated in inflammation in pancreatic islets [58], in adipose tissue, and in endothelial cells [65]. These pathways appear to operate both locally (e.g., periodontal tissue) and systemically, mediating crosstalk among tissues.

#### 3.4.3. The Oral–Gut–Systemic Axis

Beyond the local effects of oral inflammation, emerging data suggest a more complex microbiome-mediated axis that links the oral cavity, gut, and systemic circulation. Dysbiosis of the oral microbiota—characterized by overgrowth of pathogens such as *P. gingivalis* and *F. nucleatum*—has been shown to influence gut microbial composition and promote intestinal permeability (“leaky gut”), which facilitates systemic endotoxemia [66].

A recent study found that periodontitis altered gut microbial diversity and abundance in patients with diabetes, particularly increasing the abundance of pro-inflammatory Enterobacteriaceae species [42]. These alterations can amplify insulin resistance and systemic inflammation, contributing to the pathogenesis of both CVD and metabolic disease [67,68].

#### 3.4.4. Genetic and Epigenetic Links

Recent advances in genomics and epigenomics have uncovered shared genetic predispositions. For example, polymorphisms in *CDKN2A/B*, Peroxisome Proliferator-Activated Receptor Gamma (*PPARG*), and *IL6* genes have been implicated in both DM and CVD risk and have also shown associations with severe periodontitis [51].

In a Mendelian randomization study, genetically determined periodontitis was causally associated with increased risk of ischemic heart disease in patients with diabetes, suggesting a potential genetic mediation in this triad [69].

Furthermore, shared epigenetic mechanisms, including methylation changes in inflammatory gene promoters (e.g., IL6, TNF, and ICAM1), histone acetylation/deacetylation, and altered microRNA expression, have been reported in patients with PD, DM, and CVD. For example, Kang et al. [69]. demonstrated distinct DNA methylation and histone modification profiles in periodontitis patients with type 2 diabetes, while Patrick et al. [50]. identified shared genetic and epigenetic risk factors between chronic inflammatory diseases and coronary artery diseases. These findings provide evidence that epigenetic regulation contributes to the persistence and co-progression of these conditions despite conventional therapy.

#### 3.4.5. Clinical Clustering and Risk Synergy

Patients with comorbid diabetes and periodontitis are significantly more likely to develop cardiovascular complications than those with only one condition. In a longitudinal study, the presence of both diabetes and periodontitis was associated with a 3.1-fold higher risk of myocardial infarction and a 2.7-fold higher risk of stroke compared to those without either condition [12]. Importantly, these findings highlight that the elevated cardiovascular risk is not attributable to diabetes or periodontitis alone, but to their synergistic interaction, whereby chronic hyperglycemia and periodontal inflammation jointly amplify systemic cytokine release, endothelial dysfunction, and atherosclerotic progression [7,12].

This synergistic effect highlights the importance of early identification and integrated management of at-risk individuals. It also raises the potential for using PD as an early indicator or risk enhancer in cardiovascular risk stratification models for diabetic patients.

Taken together, these results demonstrate that diabetes mellitus, periodontal disease, and cardiovascular disease are linked through overlapping inflammatory, metabolic, and microbial pathways, with each axis reinforcing the others. While the evidence strongly supports these associations, their clinical and therapeutic implications require further integration and critical evaluation. These aspects are addressed in detail in the following Discussion section.

## 4. Discussion

This review highlights three well-established axes—diabetes mellitus and periodontal disease (DM–PD), periodontal disease and cardiovascular disease (PD–CVD), and diabetes mellitus and cardiovascular disease (DM–CVD)—that converge into a triangular model of shared systemic inflammation, immune dysregulation, and microbial dysbiosis. Together, these overlapping mechanisms explain why individuals with comorbid DM and PD are at substantially higher risk of cardiovascular complications compared to those with a single condition. The evidence presented in the Results demonstrates that hyperglycemia-driven inflammation, periodontal pathogen–induced bacteremia, and systemic endothelial dysfunction represent the key pathways uniting these disorders.

### 4.1. Therapeutic Implications and Multidisciplinary Management

Because gingival tissues, pancreatic islets, adipose depots, and the vascular endothelium all represent active inflammatory sites in DM, PD, and CVD, integrated management must address both local and systemic inflammation. The convergence of DM, PD, and CVD is mediated not simply by systemic inflammation but by overlapping molecular circuits, particularly NF-κB, NLRP3 inflammasome activation, and IL-1β signaling. These pathways explain how periodontal dysbiosis, hyperglycemia, and vascular stress converge on a common axis of endothelial dysfunction. Therapeutically, interventions that target these circuits—such as IL-1β inhibitors, statins with NF-κB–modulating activity, or experimental inflammasome modulators—represent promising avenues for cross-disease management. Endothelial dysfunction, in particular, emerges as a unifying pathway, explaining how localized oral infection can amplify cardiovascular risk in susceptible diabetic patients.

Given that endothelial tissue emerges as the most critical site of convergence, therapies that reduce systemic inflammation (e.g., statins, SGLT2 inhibitors, GLP-1 receptor agonists) or directly target endothelial activation have particular translational relevance.

The interwoven pathophysiology of diabetes mellitus (DM), periodontal disease (PD), and cardiovascular disease (CVD) demands a paradigm shift in how these conditions are managed. Rather than treating each in isolation, an integrated, multidisciplinary model is emerging—one that aligns oral health with systemic disease prevention and management. This approach holds promise for improved patient outcomes and reduced healthcare burdens.

#### 4.1.1. Periodontal Therapy: Beyond Oral Health

Periodontal therapy, particularly nonsurgical interventions such as scaling and root planing (SRP), has demonstrated systemic benefits. Multiple meta-analyses have confirmed that effective periodontal treatment leads to modest but statistically significant reductions in HbA1c (−0.3% to −0.6%) in patients with type 2 diabetes [70].

Moreover, improvements in endothelial function have been documented after intensive periodontal therapy. A randomized trial showed that endothelial function, as measured by brachial artery flow-mediated dilation (FMD), improved significantly four weeks after treatment in patients with moderate-to-severe periodontitis [17].

These findings support the idea that periodontal treatment is not merely a local intervention but a systemic anti-inflammatory strategy, especially for individuals with cardiometabolic comorbidities.

#### 4.1.2. Anti-Inflammatory and Cardiometabolic Medications

Sodium–glucose cotransporter-2 (SGLT2) inhibitors are a class of oral antidiabetic agents that lower blood glucose by promoting urinary glucose excretion and have demonstrated robust benefits in reducing heart failure hospitalization and cardiovascular mortality in type 2 diabetes [71,72,73]. Glucagon-like peptide-1 (GLP-1) receptor agonists are injectable incretin-based therapies that enhance glucose-dependent insulin secretion, delay gastric emptying, and promote weight reduction; beyond glycemic control, large cardiovascular outcome trials such as LEADER and SUSTAIN-6 have shown significant reductions in major adverse cardiovascular events [46,74,75,76]. These pharmacological backgrounds provide important context for understanding why SGLT2 inhibitors and GLP-1 receptor agonists may also exert beneficial effects on periodontal inflammation and systemic inflammatory pathways.

Drugs that reduce systemic inflammation and improve metabolic profiles may also benefit periodontal health:

- SGLT2 inhibitors (e.g., empagliflozin, dapagliflozin) and GLP-1 receptor agonists (e.g., liraglutide, semaglutide) have been shown to reduce systemic inflammation and oxidative stress, which could theoretically benefit periodontal tissues [17].
- Statins, primarily used for lipid-lowering and cardiovascular protection, possess anti-inflammatory and antibacterial properties. It was reported that statin use was associated with reduced periodontitis progression, particularly in patients with elevated hs-CRP [17,77].
- Low-dose doxycycline, a Matrix Metalloproteinase (MMP)-inhibitor, has been U.S. Food and Drug Administration (FDA)-approved as an adjunctive periodontal therapy and shows promise in reducing systemic inflammatory burden, including CRP and IL-6 levels [78].

Recent evidence indicates that certain pharmacological therapies may exert synergistic effects when combined with periodontal treatment. For example, statin therapy, in addition to lowering lipids and reducing cardiovascular risk, has been shown to enhance the outcomes of nonsurgical periodontal therapy by attenuating periodontal inflammation and reducing probing depth progression [33,77]. Similarly, GLP-1 receptor agonists and SGLT2 inhibitors have been validated in cardiovascular outcome trials [40,45,48], also reduce systemic inflammation and oxidative stress, which may indirectly benefit periodontal health. These data suggest that combining periodontal therapy with cardiometabolic drugs could provide additive anti-inflammatory benefits.

These pharmacologic strategies highlight the potential for cross-benefits when targeting systemic inflammation.

#### 4.1.3. Probiotics and Microbiome Modulation

Given the emerging role of oral and gut microbiota in the pathogenesis of DM, PD, and CVD, microbiome-based therapies are under investigation. Probiotic lozenges containing *Lactobacillus reuteri* have demonstrated some efficacy in reducing periodontal inflammation [79].

Pilot studies are also examining whether modulating the gut microbiome in diabetic patients with prebiotic fibers or probiotic supplements could reduce systemic endotoxemia and improve oral and cardiovascular health. While results are preliminary, this area holds potential for integrative and preventive medicine [80].

#### 4.1.4. Lifestyle Interventions: Diet, Exercise, and Smoking Cessation

Lifestyle modifications remain foundational. Nutritional approaches like the Mediterranean diet and DASH diet have been shown to improve glycemic control, reduce cardiovascular risk, and lower inflammatory biomarkers, while also improving periodontal parameters [81,82].

Beyond pharmacological approaches, nutritional interventions may work synergistically with periodontal therapy and systemic treatments. The Mediterranean and DASH diets, rich in fruits, vegetables, whole grains, polyphenols, and omega-3 fatty acids, have been shown to improve glycemic control, reduce systemic inflammation, and lower cardiovascular risk, while also improving periodontal parameters [36]. Polyphenol supplementation has demonstrated modulatory effects on periodontal bacteria and matrix metalloproteinase activity, providing an additional anti-inflammatory pathway that may complement both systemic drugs and local periodontal therapies [70]. These findings highlight the potential of coordinated drug–diet–oral health strategies to achieve more robust clinical benefits. Table 1 summarizes pharmacological and dietary strategies that not only provide primary benefits in DM or CVD management but also exert synergistic effects on periodontal health through shared anti-inflammatory and metabolic pathways.

Smoking cessation is particularly critical, as smoking amplifies all three conditions. Smokers with diabetes and periodontitis are at exponentially higher risk for cardiovascular events, making cessation programs essential in any integrated care strategy [77].

#### 4.1.5. Integrated Care Models

Several models of integrated care have been proposed and tested:

The Centre for Oral-Systemic Health (United States of America) and National Health Service NHS-integrated diabetes-periodontal clinics (UK) represent institutional examples of interdisciplinary care teams including dentists, endocrinologists, cardiologists, and nutritionists [83].

A pilot program demonstrated that embedding dental professionals within diabetes management clinics led to improved oral health literacy, higher periodontal treatment uptake, and better glycemic control at 6 months [77].

These programs not only improve clinical outcomes but may also reduce long-term healthcare costs through prevention and early intervention.

#### 4.1.6. Digital Health and Artificial Intelligence (AI)

AI-powered risk stratification tools and telemedicine platforms are beginning to play a role in identifying patients at the intersection of DM, PD, and CVD. Machine learning models trained on electronic health record (EHR) data have successfully predicted cardiovascular events in diabetic patients using periodontal status as a variable, suggesting that oral health indicators can enhance risk models [84].

Apps that integrate oral hygiene tracking with glucose monitoring and heart rate variability analysis are also under development, supporting patient self-management across domains.

### 4.2. Limitations in Current Research and Gaps in Knowledge

Despite compelling evidence supporting the associations between diabetes mellitus (DM), periodontal disease (PD), and cardiovascular disease (CVD), several limitations persist in the current literature that constrain our ability to establish causality and implement unified care strategies.

#### 4.2.1. Heterogeneity in Study Designs and Populations

Many existing studies vary in methodology, including the diagnostic criteria for PD, definitions of glycemic control, and endpoints for cardiovascular outcomes. This heterogeneity reduces the comparability of results and weakens the ability to generate robust meta-analytic conclusions.

For example, a review noted that more than 40% of trials assessing PD and CVD failed to standardize periodontal disease classification, leading to inconsistencies in findings [85].

Additionally, many cohort studies are conducted in high-income countries, limiting generalizability to low- and middle-income populations where the burden of all three diseases is rising [86].

#### 4.2.2. Confounding and Reverse Causality

Many observational studies are prone to confounding factors such as smoking, obesity, diet, and socioeconomic status, all of which influence DM, PD, and CVD. While multivariate adjustment helps, residual confounding remains a challenge. Moreover, the bidirectional nature of the relationships—especially between DM and PD—complicates interpretation of causality.

Mendelian randomization (MR) studies are emerging as tools to overcome confounding, but even MR analyses are limited by the availability and specificity of genetic instruments [87].

#### 4.2.3. Underpowered or Short-Term Interventional Studies

Though some interventional trials suggest periodontal therapy may improve glycemic control or endothelial function, most are short (e.g., ≤6 months) and lack hard cardiovascular outcomes like myocardial infarction or stroke. A meta-analysis emphasized the need for long-term randomized controlled trials (RCTs) with clinical endpoints [30].

The absence of large-scale, longitudinal interventional studies hinders the development of evidence-based integrated treatment guidelines.

#### 4.2.4. Lack of Mechanistic Human Studies

Most mechanistic insights into inflammation, dysbiosis, or immune pathways are derived from in vitro or animal studies. While useful, these models do not fully replicate the human biological complexity. There is a need for well-designed human studies that utilize tissue biopsies, single-cell sequencing, and proteomic profiling to map real-time interactions between oral and systemic inflammation [88,89].

Addressing these limitations will be crucial to translating current evidence into actionable clinical practice. Standardization of definitions, long-term interventional data, and inclusion of diverse populations must be prioritized. Additionally, mechanistic human studies will strengthen causal inference and facilitate biomarker development. Overcoming these research gaps is essential for moving from correlation to causation, and from fragmented care to unified disease prevention.

It should also be noted that comparable inflammatory and microbial pathways are implicated in other diabetes-related complications. For example, diabetic nephropathy is characterized by endothelial dysfunction and NF-κB–driven cytokine release within renal tissues, while diabetic retinopathy involves chronic low-grade inflammation, oxidative stress, and vascular leakage in the retina [7,68,90,91]. Beyond diabetes, systemic inflammatory diseases such as rheumatoid arthritis share overlapping cytokine profiles (e.g., elevated IL-6 and TNF-α) and are associated with increased periodontal and cardiovascular risk. Recognizing these parallels situates the DM–PD–CVD triad within a broader network of chronic inflammatory conditions, highlighting the systemic relevance of these mechanisms.

### 4.3. Future Directions

Addressing current gaps will require a shift toward more integrative, precise, and equitable research frameworks. Several promising directions are emerging. Although not applied in the present review, AI-driven natural language processing and automated literature-mining platforms are emerging as valuable tools to support systematic evidence synthesis and risk stratification, and they may play an important role in future integrative analyses of DM, PD, and CVD [84,92].

#### 4.3.1. Precision Medicine and Risk Stratification

As multi-omics and machine learning tools mature, individualized risk prediction models incorporating genetic, inflammatory, microbiome, and clinical data will become feasible. Precision strategies [64] could stratify patients based on inflammatory profiles, enabling personalized periodontal and metabolic therapies.

A study described an AI-integrated model combining oral microbiome signatures with HbA1c and hs-CRP levels to predict future cardiovascular events with 85% accuracy in diabetic patients—a proof-of-concept for precision prevention [93].

#### 4.3.2. Integrated Clinical Trials

Large-scale, multicenter RCTs that evaluate the impact of combined dental and medical interventions on systemic outcomes are urgently needed. Ongoing trials and studies in Europe are evaluating whether routine periodontal therapy in diabetic patients reduces long-term CVD risk [94,95,96].

Such trials should use standardized periodontal assessment tools and include long-term follow-up for hard outcomes, enabling definitive conclusions.

#### 4.3.3. Microbiome and Metabolome Research

Future research should focus on oral-gut-systemic microbial interactions using metagenomics, metabolomics, and lipidomics. Understanding how microbial metabolites like trimethylamine-N-oxide (TMAO) or short-chain fatty acids modulate systemic inflammation could open new therapeutic avenues, including dietary or probiotic interventions [67].

#### 4.3.4. Therapeutic Targets and Emerging Mechanistic Strategies

This subsection highlights therapeutic targets that act on common molecular circuits linking DM, PD, and CVD, including pharmacological agents, microbiome-modulating therapies, nutritional bioactives, and AI-driven strategies.

New pharmacological agents such as sodium-glucose co-transporter-2 (SGLT2) inhibitors and glucagon-like peptide-1 (GLP-1) receptor agonists, already validated in large cardiovascular outcome trials, have demonstrated systemic anti-inflammatory effects and improved endothelial function. Preliminary evidence suggests that these agents may also reduce periodontal inflammation, although larger dedicated trials are needed. Similarly, statins, traditionally used for lipid lowering, have shown promise in slowing periodontal disease progression due to their anti-inflammatory and antibacterial properties [97,98].

Microbiome-modulating therapies are another emerging field. Probiotics (e.g., Lactobacillus reuteri lozenges) and prebiotics have been studied as adjuncts to periodontal therapy, with early results suggesting reductions in gingival inflammation and systemic inflammatory markers. Recent patents have also described novel anti-inflammatory and microbiome-targeted formulations, including polyphenol-based compounds and engineered bacterial strains, designed to simultaneously modulate oral and gut dysbiosis with potential benefit across DM, PD, and CVD [10,99].

Novel mechanistic insights have been provided by multi-omics analyses that integrate genomics, transcriptomics, proteomics, and metabolomics. These studies have identified shared signatures of inflammation, including IL-6, TNF-α, and NF-κB signaling, as well as alterations in lipid and amino acid metabolism, which may represent therapeutic targets. The concept of an “oral–gut–systemic axis” has gained attention, suggesting that periodontal dysbiosis can influence gut microbiota composition and systemic immune activation, thereby linking PD to both metabolic and cardiovascular dysfunction [25].

AI-driven approaches represent an additional frontier. Machine-learning models that integrate periodontal status, glycemic indices, and systemic biomarkers have shown promise in predicting cardiovascular events in diabetic populations. These tools may support personalized risk stratification and highlight oral health as a variable of clinical importance in cardiometabolic care [100,101,102].

Together, these therapeutic targets demonstrate how interventions directed at shared molecular pathways (NF-κB, NLRP3 inflammasome, IL-1β, IL-6/CRP axis) may yield synergistic benefits across DM, PD, and CVD, and should be prioritized in future translational trials.

#### 4.3.5. Policy and Practice Integration

Healthcare systems must evolve to incorporate dental care as part of chronic disease management. Policies that incentivize interdisciplinary collaboration, integrated electronic health records, and co-location of dental and medical services could significantly improve outcomes.

Moreover, educating clinicians—especially in primary care—about the oral-systemic disease connection should be prioritized in medical and dental curricula [103].

The convergence of digital health, systems biology, and interdisciplinary care is paving the way for a new era in chronic disease management. The future lies in data-driven, integrated models that detect disease earlier, intervene more precisely, and monitor more holistically. Success in this endeavor will depend not only on scientific innovation but also on policy reform, clinician education, and patient empowerment.

Taken together, these findings underscore the need to shift from a siloed approach to a truly integrated model of care. Periodontal health should be recognized as a cardiometabolic risk factor, and its management incorporated into diabetes and cardiovascular care pathways. At the same time, the convergence of novel therapies, microbiome-targeted interventions, and AI-driven precision tools offers new opportunities to reduce the inflammatory burden that unites these conditions. By embedding oral-systemic health within multidisciplinary care and policy frameworks, clinicians and researchers can help reshape prevention and management strategies for chronic diseases worldwide.

## 5. Conclusions

The evidence connecting diabetes mellitus, periodontal disease, and cardiovascular pathologies is compelling and continues to grow. These conditions, long viewed as distinct entities, are now recognized as components of a complex, interconnected disease network driven by chronic inflammation, immune dysregulation, microbial dysbiosis, and metabolic dysfunction.

Bidirectional relationships between diabetes and periodontitis, and between periodontitis and cardiovascular disease, underscore the need for a systemic view of oral health. Furthermore, diabetes itself significantly increases cardiovascular risk through a host of overlapping mechanisms, including endothelial dysfunction, oxidative stress, and pro-inflammatory states—all of which are further exacerbated by coexisting periodontitis.

Emerging research, including omics-based systems biology and advanced machine learning, is beginning to unravel the shared molecular pathways that drive this triad. Clinically, the integration of periodontal care into cardiometabolic risk management has already shown promise, with evidence supporting modest improvements in glycemic control, endothelial function, and inflammatory biomarkers.

However, significant gaps remain—particularly the lack of large, long-term interventional trials with definitive cardiovascular outcomes. Moving forward, precision medicine, integrated care models, and microbiome-focused interventions may reshape how these diseases are diagnosed, treated, and prevented.

Despite promising evidence for the systemic benefits of periodontal therapy and cardiometabolic drugs, significant gaps remain—particularly the lack of large, long-term interventional trials with definitive cardiovascular outcomes. Moving forward, multidisciplinary care models that bridge dentistry, endocrinology, and cardiology, combined with precision medicine and microbiome-targeted therapies, will be essential to reduce the global burden of these interconnected conditions and improve long-term outcomes.

## Figures and Tables

**Table 1:** Examples of pharmacological and nutritional strategies with synergistic effects across DM, PD, and CVD.

Intervention	Primary Clinical Effect	Additional/Synergistic Effect on DM–PD–CVD Axis	References
Statins	Lipid-lowering, cardiovascular protection	Reduce periodontal inflammation and disease progression; enhance outcomes of nonsurgical periodontal therapy	[33,77]
SGLT2 inhibitors (e.g., empagliflozin, dapagliflozin)	Improve glycemic control, reduce heart failure risk and CV mortality	Anti-inflammatory and antioxidative effects may benefit periodontal health	[40,41,48]
GLP-1 receptor agonists (e.g., liraglutide, semaglutide)	Improve glycemic control, reduce MACE in T2DM	Reduce systemic inflammation and improve endothelial function; potential positive effects on PD outcomes	[44,45,46]
Polyphenols (e.g., resveratrol, flavonoids)	Antioxidant and anti-inflammatory dietary compounds	Modulate periodontal bacteria and matrix metalloproteinase (MMP) activity; support metabolic and vascular health	[70]
Omega-3 fatty acids (e.g., EPA, DHA)	Improve lipid profile, reduce CV risk	Enhance resolution of periodontal inflammation; improve insulin sensitivity	[36]
Mediterranean/DASH diet	Improve glycemic control, lower blood pressure, reduce CV events	Reduce periodontal inflammation, improve periodontal indices, and lower systemic inflammatory markers	[36]

Pharmacological and nutritional interventions with synergistic effects across diabetes mellitus (DM), periodontal disease (PD), and cardiovascular disease (CVD). The table highlights how drugs and dietary strategies provide their primary benefits while simultaneously exerting secondary effects that reduce systemic inflammation, improve periodontal outcomes, and mitigate cardiovascular risk. Abbreviations: CVD—cardiovascular disease; CV—Cardiovascular; DASH—Dietary Approaches to Stop Hypertension; DM—Diabetes Mellitus; EPA—Eicosapentaenoic Acid; DHA—Docosahexaenoic Acid; GLP-1—Glucagon-Like Peptide-1; MACE—Major Adverse Cardiovascular Events; MMP—Matrix Metalloproteinase; NSPT—Nonsurgical Periodontal Therapy; PD—Periodontal Disease; SGLT2—Sodium-Glucose Cotransporter-2; T2DM—Type 2 Diabetes Mellitus.

## Data Availability

No new data were created or analyzed in this study. Data sharing does not apply to this article.

## Abbreviations

The following abbreviations are used in this manuscript:

AI	Artificial Intelligence
CAD	Coronary Artery Disease
CRP	C-Reactive Protein
CVD	Cardiovascular Disease
DAPA-HF	Dapagliflozin and Prevention of Adverse Outcomes in Heart Failure
DASH	Dietary Approaches to Stop Hypertension
DM	Diabetes Mellitus
DNA	Deoxyribonucleic Acid
EHR	Electronic Health Record
EMPA-REG	Empagliflozin Cardiovascular Outcome Event Trial in Type 2 Diabetes Mellitus Patients—Removing Excess Glucose
FDA	U.S. Food and Drug Administration
FMD	Flow-Mediated Dilation
GLP	Glucagon-Like Peptide
HDL	High-Density Lipoprotein
ICAM	Intercellular Adhesion Molecule
IL	Interleukin
LEADER	Liraglutide Effect and Action in Diabetes: Evaluation of Cardiovascular Outcome Results
LPS	Lipopolysaccharide
MACE	Major Adverse Cardiovascular Events
MI	Myocardial Infarction
MMP	Matrix Metalloproteinase
MR	Mendelian Randomization
NF	Nuclear Factor
NHS	National Health Service
NSPT	Nonsurgical Periodontal Therapy
OUTCOME	Outcome Trial (e.g., EMPA-REG OUTCOME)
PD	Periodontal Disease
PPARG	Peroxisome Proliferator-Activated Receptor Gamma
RAGE	Receptor for Advanced Glycation End-products
ROS	Reactive Oxygen Species
SRP	Scaling and Root Planing
SUSTAIN	Semaglutide Unabated Sustainability in Treatment of Type 2 Diabetes
TMAO	Trimethylamine N-oxide
TNF	Tumor Necrosis Factor
UK	United Kingdom
U.S.	United States of America
VCAM	Vascular Cell Adhesion Molecule
VLDL	Very-Low-Density Lipoprotein
